# Transmission of Vancomycin-Resistant Enterococci in the Hospital Setting: Uncovering the Patient–Environment Interplay

**DOI:** 10.3390/microorganisms8020203

**Published:** 2020-01-31

**Authors:** Carlos L. Correa-Martinez, Hauke Tönnies, Neele J. Froböse, Alexander Mellmann, Stefanie Kampmeier

**Affiliations:** 1 Institute of Hygiene, University Hospital Münster, Robert-Koch-Straße 41, 48149 Münster, Germany; Carlos.Correa@ukmuenster.de (C.L.C.-M.); Hauke.Toennies@ukmuenster.de (H.T.); Alexander.Mellmann@ukmuenster.de (A.M.); 2 Institute of Medical Microbiology, University Hospital Münster, Domagkstraße 10, 48149 Münster, Germany; Neele.Froboese@ukmuenster.de

**Keywords:** VRE, environment, contamination, infection, transmission, whole-genome sequencing

## Abstract

Vancomycin-resistant enterococci (VRE) are relevant nosocomial pathogens with an increasing incidence in the last decades. Their transmission is optimal in the hospital setting, as it offers two potential, large reservoirs that are closely related: susceptible patients and their environment. Here we investigate the role of the hospital environment in the nosocomial transmission of VRE by establishing concrete links between contaminated surfaces and colonized/infected patients in outbreak and non-outbreak settings. Environmental and patient VRE isolates were collected between 2013 and 2019 and analyzed by whole-genome sequencing (WGS), subsequent multilocus sequence typing (MLST), and core genome (cg) MLST. Pairs of isolates differing in <3 alleles were rated as closely related, making a transmission likely. Fifty-three environmental VRE isolates were analyzed. MLST sequence types (ST) ST203 (50.0%), ST192 (21.3%), ST117 (17.3%), ST721 (8.8%), ST80 (2%), and ST1489 (0.7%) were detected, carrying the resistance determinants *vanA* (72.7%), *vanB* (24%), or both (3.3%). Of the 53 environmental isolates, 51 were found to form five clusters with genetically related patient isolates (*n* = 97 isolates). WGS confirms the role of the environment in the transmission dynamics of VRE in both the outbreak and non-outbreak settings, highlighting the importance of prevention and control of VRE spread.

## 1. Introduction

Vancomycin-resistant enterococci (VRE) are Gram-positive microorganisms that significantly contribute to the burden of healthcare-associated infections. First reported in Europe in the late 1980s [1,2], VRE are currently considered a public health problem, regarded as a high-level priority for the research and development of new therapeutic strategies by the World Health Organization [3]. The incidence of VRE infections has experienced a steady increase in different regions in the last decades [4], a trend also observed in Germany [5]. Considering the significantly higher mortality rate of VRE infections as compared to those caused by vancomycin-susceptible enterococci [6,7], and in view of the well-documented inter- and intra-species transmission of resistance genes [8,9], it has become necessary to adopt infection control strategies worldwide aimed at reducing the spread of VRE [10]. The clinical and epidemiological impact of VRE on the severity and burden of enterococcal disease [4,6,7], points out the relevance of identifying and understanding the conditions favoring the development and transmission of VRE in order to prevent their spread and ultimately reduce the incidence of infections. The healthcare setting represents the main scenario for the implementation of such strategies, given that it offers optimal conditions for the transmission and spread of multidrug-resistant microorganisms.

VRE are more frequently involved in nosocomial transmission events than other relevant resistant bacteria [11]. Hospitalized patients constitute an important element in the transmission dynamics of VRE, representing large reservoirs of susceptible individuals often displaying characteristics known to be associated with an increased risk of VRE colonization and infection, such as immunosuppression, multi-morbidity, and treatment with antimicrobial substances [12,13,14]. Furthermore, the colonization pressure exerted by VRE-positive patients on the susceptible population [15,16], as well as the close and constant interaction between staff members and patients, [17] facilitates transmission. A second key element in the nosocomial spread of VRE is the environment, comprising objects and surfaces that may act as potential reservoir [16,18]. VRE are known to attach to different materials and persist for up to several years on environmental surfaces [18]. Moreover, it has been observed that room contamination persists after the application of standard cleaning procedures following discharge of VRE-positive patients, leading to a significantly higher risk of VRE acquisition for new patients assigned to these rooms [16,19]. Elements of the environment have been identified as the source of outbreaks in the past [20]. However, demonstrating transmission chains by matching findings of environmental samplings to specific cases of colonization and/or infection is not always possible [21,22].

In the present study, we use whole-genome sequencing (WGS)-based typing to analyze genetic relationships between VRE originating from patients and the hospital environment at a university hospital, in order to gain a deeper understanding of possible VRE transmission during outbreak and non-outbreak settings.

## 2. Materials and Methods

### 2.1. Clinical Setting and Infection Control Measures

The University Hospital Münster (UHM) is a 1500-bed tertiary care center, admitting ca. 65,000 patients per year. Routinely, in accordance with the national German guideline published in 2018 [23], patients at high risk for developing VRE infections (e.g., hemato-oncological, orthopedic, and intensive-care unit patients), patients with a VRE history of colonization/infection, or patients with contact to a patient with confirmed VRE status are routinely screened for VRE. In case of VRE detection, affected patients are isolated in a separate room and contact precautions are established. Personnel staff and visitors are advised to wear gloves and gowns when entering the patient’s room. Surface disinfection using Incidin ^TM^ plus 0.5% (ECOLAB Healthcare, Monheim am Rhein, Germany) soaked wipes is performed at least once a day. Patient isolation can be discontinued if three negative anorectal swab samples are collected in at least a one-week interval without administering antibiotic therapy during this time.

### 2.2. Environmental Surveillance

At the UHM, environmental sampling is performed routinely during on-site infection control ward rounds (see also Supplementary Table S1), as well as in the case of increased incidence of multidrug-resistant bacteria or outbreaks, in which inanimate surfaces serve as possible sources of transmission. If multidrug-resistant bacteria (e.g., Methicillin resistant *Staphylococcus aureus* (MRSA), VRE, or multidrug-resistant Gram-negative bacteria) are detected, WGS-based typing methods are performed routinely to elucidate transmission chains at an early stage [24].

For the present study, all sequenced environmental isolates detected between January 2013 and August 2019 were retrospectively compared to all VRE strains isolated from patients admitted to the UHM during the same time period.

### 2.3. Sampling and Testing Methods

Screening and clinical samples were sent to the microbiological laboratory. Depending on the specimen the sample was streaked onto different agar media (e.g., Columbia sheep blood agar, Oxoid, Wesel, Germany and/or VRE selective agar, VRESelect, Biorad, Hercules, CA, USA) and incubated for 18 ± 2 h at 37 °C. Bacterial species of suspected colonies were identified by MALDI-TOF-MS (Bruker Corporation, Bremen, Germany) and antibiotic susceptibility testing was performed using VITEK®2 system (BioMérieux, Nürtingen, Germany) in accordance with the European Committee on Antimicrobial Susceptibility Testing (EUCAST) standards for clinical breakpoints [25]. The GenoType Enterococcus system (Hain Lifescience, Nehren, Germany) was used to differentiate vancomycin resistance genes *vanA* and *vanB*. Subsequently, isolates were whole-genome sequenced.

Environmental sampling was performed by applying sterile packaged polywipes (MWE, Corsham, Wiltshire, UK) on hand contact surfaces, which were incubated in Tryptic Soy Broth + lecithin Tween (LT) (Merck Millipore, Eppelheim, Germany) for 24 h at 37 °C. Ten microliters of this broth was streaked onto blood agar and VRE selective agar and incubated for 24 h at 37 °C. Suspected colonies were subcultured on blood agar and species identification was performed with the help of MALDI-TOF-MS (Bruker Corporation). Susceptibility testing for vancomycin was performed using Etest® (Bestbion GmbH, Liofilchem, Italy) and evaluated in accordance with the EUCAST standards for clinical breakpoints [25]. Confirmed VRE were whole-genome sequenced.

### 2.4. Whole-Genome Sequencing

Confirmed VRE isolates were subjected to WGS using the Illumina MiSeq or HiSeq platform (Illumina Inc., San Diego, CA, USA). After sequencing, quality trimming, and de novo assembly, coding regions were compared in a gene-by-gene approach, i.e., core genome multilocus sequence typing (cgMLST) using the SeqSphere+ software version 6.0.0 (Ridom GmbH, Muenster, Germany) as described previously. For cgMLST analysis, the public cgMLST scheme for *Enterococcus faecium* was applied [26]. In order to elucidate genetic relationships between isolates, a minimum spanning tree algorithm was applied using the same software. MLST sequence types were extracted in silico.

## 3. Results

### 3.1. Environmental and Patient VRE Isolates

From October 2013 to August 2019, in total, 53 environmental samples containing VRE were identified. During the retrospective investigation, 97 VRE patient isolates of patients admitted to the UHM during this time span could be detected as being genetically related to these environmental isolates. Further information regarding date of isolation and sampling point can be derived from Supplementary Table S1.

### 3.2. VRE Genotypes and Genetic Distribution of Strains

**Figure 1:**
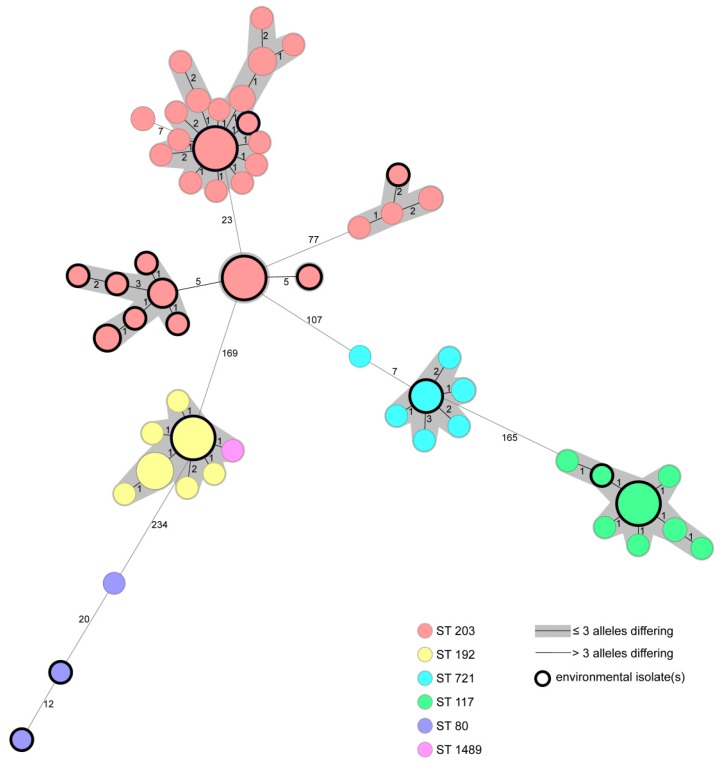
Minimum spanning tree of 150 VRE strains isolated from patients or environmental sampling points based on 1423 core genome multilocus sequence typing (cgMLST) target genes, pairwise ignore missing values. Every circle represents one genotype, the size of circles correlates with the number of identical genotypes. Different colors represent different MLST sequence types. Black bordered dots indicate environmental isolates. Grey coloring indicates close genetic relation (≤3 alleles differing between two genotypes).

WGS resulted in four singletons (two patient and two environmental isolates) and eight clusters of genetically closely related strains (≤3 alleles differing between the genotypes) comprising 2–20 isolates. Of these, five clusters contained environmental and patient isolates (Figure 1). Except four environmental isolates, all other isolates gathered from the environment were closely related to patient isolates collected during routine screening and within outbreak settings. Additional information of exact sample sites can be gathered from Supplementary Table S1. Of all strains, 109 (72.7%) harbored *vanA*, 36 (24%) *vanB*, and 5 (3.3%) both *vanA* and *vanB*. Prevalent MLST sequence types (ST) were ST203 (50.0%), ST192 (21.3%), ST117 (17.3%), ST721 (8.8%), ST80 (2%), and ST1489 (0.7%). Additional information of distribution of *van* genes and MLST ST in patient and environmental isolates is displayed in Table 1.

## 4. Discussion

In the present study, we investigated the role of the contaminated hospital environment and its impact on VRE transmission, considering that these microorganisms are able to survive on inanimate surfaces for several years [18]. The analysis and comparison of VRE isolates obtained from environmental surfaces and patients revealed distinct genetic relationships between both groups of isolates, confirming that the environment acts as a suitable reservoir that can facilitate the spread of VRE and, thus, the subsequent occurrence of infections. The development of vancomycin-resistant bacterial populations in association with antibiotic treatment has been described in enterococci [27]. In addition, environmental contamination has been described to play a role in the nosocomial transmission of multidrug-resistant microorganisms [18,28]. Subsequently, resistant strains are transmitted via direct (person–person) or indirect (person–environment–person) contact [28]. Both events commonly occur in the healthcare setting, where VRE have shown to be more commonly transmitted than other relevant multidrug-resistant pathogens [11]. A limitation of our study is that we cannot conclude whether VRE was transmitted from surfaces to patients or vice versa. However, it seems most plausible that this microorganism transfer is a two-way process. The occurrence of the same genotype in patient and environmental isolates at least highlights two important aspects: 1) Person–environment transmission of VRE plays a role in everyday clinical practice and 2) aurface disinfection techniques during clinical routine have to be adequate to prevent person–environment transmissions.

## 5. Conclusions

Inanimate surfaces in the hospital environment are a relevant source of transmission of VRE during clinical routine. Improving surface disinfection procedures of possibly VRE-contaminated rooms is an important aspect in preventing VRE infections. Further prospective studies are needed to demonstrate direct links between contaminated surfaces and patients colonized or infected with VRE.

## Figures and Tables

**Table 1:** Distribution of *van* genes and multilocus sequence typing (MLST) sequence types (ST) in detected vancomycin-resistant enterococci (VRE) isolates derived from patients (P) and the hospital environment (E).

	MLST ST						*van* genotype		
	ST80	ST117	ST192	ST203	ST721	ST1489	*van*A	*van*B	*van*A+ *van*B
P (*n* = 97)	1 (1.0%)	21 (21.6%)	24 (24.7%)	39 (40.2%)	11 (11.3%)	1 (1.0%)	67 (69.1%)	26 (26.8%)	4 (4.1%)
E (*n* = 53)	2 (3.8%)	5 (9.4%)	8 (15.1%)	36 (67.9%)	2 (3.8%)	0 (0%)	42 (79.2%)	10 (18.9%)	1 (1.9%)

## Supplementary Materials

The following are available online at https://www.mdpi.com/2076-2607/8/2/203/s1, Table S1: MLST sequence type (ST), collection date and localization of 150 chronologically listed VRE isolates derived from patients (P) and the hospital environment (E).
