# Supplementary material for: Transmission of Vancomycin-Resistant Enterococci in the Hospital Setting: Uncovering the Patient–Environment Interplay
Source: Microorganisms. 2020 Jan 31;8(2):203. doi: 10.3390/microorganisms8020203 (PMC7074967; doi:10.3390/microorganisms8020203)
Supplement: Supplementary file 1 [file microorganisms-08-00203-s001.pdf]

microorganisms

MDPI

Supplementary Table S1. MLST sequence type (ST), collection date and localization of 150 chronologically listed VRE isolates derived from patients (P) and the hospital environment (E).

Isolate no.	MLST ST	*van*-gene	Collection date	Localisation	Ward no.[1]
P1	80	*vanB*	2013/10/23	rectal swab	I
P2	192	*vanB*	2014/7/8	rectal swab	II
P3	192	*vanB*	2014/8/18	rectal swab	I
P4	192	*vanB*	2014/9/9	intra-abdominal swab	III
P5	192	*vanB*	2014/9/29	rectal swab	IV
P6	192	*vanB*	2014/10/12	rectal swab	III
P7	192	*vanB*	2014/10/20	rectal swab	IV
P8	192	*vanB*	2014/11/14	intra-abdominal swab	III
P9	192	*vanB*	2015/1/20	blood culture	V
P10	192	*vanB*	2015/4/4	rectal swab	I
P11	192	*vanB*	2015/6/8	rectal swab	V
P12	192	*vanB*	2015/6/9	rectal swab	V
P13	192	*vanB*	2015/6/9	rectal swab	V
P14	192	*vanB*	2015/6/16	rectal swab	VI
P15	192	*vanB*	2015/6/19	rectal swab	V
P16	192	*vanB*	2015/6/23	rectal swab	VI
P17	192	*vanB*	2015/7/7	rectal swab	V
P18	192	*vanB*	2015/7/16	rectal swab	V
P19	192	*vanB*	2015/7/21	rectal swab	V
P20	1489	*vanB*	2015/7/30	rectal swab	V
P21	192	*vanB*	2015/8/6	rectal swab	V
P22	192	*vanB*	2015/8/6	rectal swab	V
P23	192	*vanB*	2015/8/20	intra-abdominal swab	VII
P24	192	*vanB*	2016/2/2	rectal swab	VIII
P25	117	*vanA*	2016/2/24	rectal swab	III
P26	117	*vanA*	2016/3/3	intra-abdominal swab	VII
P27	117	*vanA*	2016/3/23	stool sample	IX
P28	117	*vanA*	2016/3/26	rectal swab	X
P29	117	*vanA*	2016/3/27	stool sample	XI
P30	117	*vanA*	2016/4/12	urine culture	X
P31	117	*vanA*	2016/4/16	blood culture	X
P32	117	*vanA*	2016/4/26	rectal swab	VI
P33	117	*vanA*	2016/4/26	urine culture	I
P34	117	*vanA*	2016/5/12	rectal swab	VI
P35	117	*vanA*	2016/5/12	rectal swab	VI
P36	117	*vanA*	2016/5/13	rectal swab	VI
P37	192	*vanB*	2016/5/15	urine culture	VI
P38	117	*vanA*	2016/5/17	stool sample	XII
P39	117	*vanA*	2016/6/9	stool sample	XIII

P40	117	*vanA*	2016/6/9	rectal swab	VI
P41	203	*vanA*	2016/6/11	intra-abdominal swab	XIV
P42	117	*vanA*	2016/6/13	rectal swab	V
P43	117	*vanA*	2016/6/24	rectal swab	V
P44	203	*vanA*	2016/6/28	rectal swab	V
P45	203	*vanA*	2016/6/30	rectal swab	XV
P46	203	*vanA*	2016/8/3	rectal swab	XVI
P47	203	*vanA, vanB*	2016/8/26	stool sample	XVII
P48	203	*vanA*	2016/8/26	stool sample	III
P49	117	*vanA*	2016/9/3	rectal swab	I
P50	117	*vanA*	2016/9/5	rectal swab	VI
P51	203	*vanA*	2016/9/5	rectal swab	XVIII
P52	203	*vanA*	2016/9/22	tissue	XVIII
P53	203	*vanA*	2016/9/26	rectal swab	XVIII
P54	203	*vanA*	2016/9/27	rectal swab	XI
P55	203	*vanA*	2016/9/27	rectal swab	XVI
P56	117	*vanA*	2016/9/28	rectal swab	XVIII
P57	203	*vanA*	2016/10/2	rectal swab	XIX
P58	117	*vanA*	2016/10/8	rectal swab	IV
P59	203	*vanA*	2016/10/12	stool sample	XX
P60	203	*vanA*	2016/10/17	rectal swab	XVI
P61	203	*vanA*	2016/10/18	rectal swab	XXI
P62	203	*vanA*	2016/10/19	rectal swab	XI
P63	203	*vanA*	2016/10/27	rectal swab	XXII
P64	203	*vanA*	2016/10/27	rectal swab	XXII
P65	203	*vanA*	2016/11/7	rectal swab	XXI
P66	203	*vanA*	2016/11/14	rectal swab	XVIII
P67	203	*vanA*	2016/11/23	rectal swab	VI
P68	203	*vanA, vanB*	2016/11/29	rectal swab	XI
P69	203	*vanA, vanB*	2016/12/5	rectal swab	XI
P70	203	*vanA, vanB*	2017/1/17	rectal swab	XVII
P71	203	*vanA*	2017/2/5	rectal swab	XVIII
P72	203	*vanA*	2017/2/7	rectal swab	XI
P73	203	*vanA*	2017/2/7	rectal swab	XI
P74	203	*vanA*	2017/3/13	rectal swab	XVIII
P75	203	*vanA*	2017/3/13	rectal swab	XVIII
P76	203	*vanA*	2017/3/15	rectal swab	XI
P77	203	*vanA*	2017/3/19	stool sample	XXIII
P78	203	*vanA*	2017/4/3	rectal swab	XVII
P79	203	*vanA*	2017/4/17	rectal swab	IV
P80	192	*vanB*	2017/6/7	rectal swab	VI
P81	721	*vanA*	2019/7/1	urine culture	XXIV
P82	721	*vanA*	2019/7/2	dialysis catheter	III

P83	721	*vanA*	2019/7/3	rectal swab	XIV
P84	721	*vanA*	2019/7/5	urine culture	XXV
P85	721	*vanA*	2019/7/6	rectal swab	III
P86	721	*vanA*	2019/7/8	rectal swab	XIV
P87	721	*vanA*	2019/7/19	stool sample	XXIV
P88	721	*vanA*	2019/7/20	stool sample	XXVI
P89	721	*vanA*	2019/7/26	rectal swab	XXVII
P90	203	*vanA*	2017/8/14	rectal swab	XVII
P91	721	*vanA*	2019/8/24	stool sample	XXVI
P92	721	*vanA*	2019/8/28	stool sample	XXVIII
P93	203	*vanA*	2017/10/30	rectal swab	XVI
P94	203	*vanA*	2017/10/30	rectal swab	XVI
P95	203	*vanA*	2017/11/6	rectal swab	XVI
P96	203	*vanA*	2017/11/13	rectal swab	XVI
P97	203	*vanA*	2017/11/30	rectal swab	XXIX
E1	203	*vanA*	2013/12/13	wheelchair	III
E2	203	*vanA*	2013/12/13	walking frame	III
E3	203	*vanA*	2013/12/30	nursing staff centre	X
E4	203	*vanA*	2013/12/30	remote control	X
E5	192	*vanB*	2015/5/11	first aid cabinet	VI
E6	192	*vanB*	2015/5/11	washing room	VI
E7	192	*vanB*	2015/5/11	disposal room	VI
E8	192	*vanB*	2015/5/11	first aid cabinet	VI
E9	192	*vanB*	2015/5/11	nursing staff centre	VI
E10	192	*vanB*	2015/5/19	commode chair	VI
E11	203	*vanA*	2015/5/27	nursing staff centre	III
E12	203	*vanA*	2015/5/27	monitor screen	III
E13	203	*vanA*	2015/5/27	patient file	III
E14	203	*vanA*	2015/5/27	first aid cabinet	III
E15	203	*vanA*	2015/5/27	infusion stand	III
E16	203	*vanA*	2015/5/27	infusion stand	III
E17	203	*vanA*	2015/5/27	sterile stocks	III
E18	203	*vanA*	2015/5/27	emergency trolley	III
E19	203	*vanA*	2015/5/27	nursing staff centre	III
E20	203	*vanA*	2015/5/27	ECG monitor	III
E21	203	*vanA*	2015/5/27	first aid cabinet	III
E22	203	*vanA*	2015/5/27	patient file	III
E23	192	*vanB*	2015/6/2	medicine cabinet	VI
E24	192	*vanB*	2015/6/2	nursing staff centre	V
E25	80	*vanB*	2015/6/2	door pull, patient room	VI
E26	80	*vanB*	2015/6/2	light switch, patient room	VI
E27	117	*vanA*	2016/4/28	air lock door	X
E28	117	*vanA*	2016/4/28	first aid cabinet	X

E29	117	*vanA*	2016/4/28	medicine cabinet	X
E30	117	*vanA*	2016/4/28	infusion stand	X
E31	117	*vanA*	2016/9/9	door pull, patient room	XVIII
E32	203	*vanA*	2016/9/9	light switch, patient room	XVIII
E33	203	*vanA*	2016/9/9	patient's bed	XVIII
E34	203	*vanA*	2016/9/9	light switch, patient room	XVIII
E35	203	*vanA*	2016/9/9	recreation room	XVIII
E36	203	*vanA*	2016/9/9	medicine cabinet	XVIII
E37	203	*vanA*	2016/9/9	medicine cabinet	XVII
E38	203	*vanA*	2016/9/9	wheelchair	XVII
E39	203	*vanA*	2016/9/9	walking frame	XVII
E40	203	*vanA*	2016/9/9	air lock door	XVII
E41	203	*vanA*	2016/9/9	heater	XVII
E42	203	*vanA*	2016/9/9	computer keyboard	XVII
E43	203	*vanA*	2016/10/4	patient file	XVIII
E44	203	*vanA*	2016/10/4	patient file	XVIII
E45	203	*vanA*	2016/10/4	dispenser	XVIII
E46	203	*vanA*	2016/10/4	infusion stand	XVIII
E47	203	*vanA*	2016/10/4	bedside table	XVII
E48	203	*vanA*	2016/10/8	recreation room	XVII
E49	203	*vanA*	2016/10/10	air lock door	XVIII
E50	203	*vanA*	2016/10/10	nursing staff centre	XVIII
E51	203	*vanA*, *vanB*	2016/10/10	nursing staff centre	XVIII
E52	721	*vanA*	2019/8/5	ventilator	III
E53	721	*vanA*	2019/8/5	medicine cabinet	III

[1] Identical numbers indicate detection of VRE on the same ward.
